# Implementation of a national rapid prenatal exome sequencing service in England: evaluation of service outcomes and factors associated with regional variation

**DOI:** 10.3389/fgene.2024.1485306

**Published:** 2024-11-06

**Authors:** Rema Ramakrishnan, Corinne Mallinson, Steven Hardy, Jennifer Broughan, Maisie Blyth, Gabriella Melis, Catherine Franklin, Melissa Hill, Rhiannon Mellis, Wing Han Wu, Stephanie Allen, Lyn S. Chitty, Marian Knight, EXPRESS Clinical Outcomes Group, Ruth Armstrong

**Affiliations:** ^1^ National Perinatal Epidemiology Unit, University of Oxford, Oxford, United Kingdom; ^2^ National Disease Registration Service, National Health Service England, London, United Kingdom; ^3^ NHS North Thames Genomic Laboratory Hub, Great Ormond Street Hospital for Children NHS Foundation Trust, London, United Kingdom; ^4^ Genetics and Genomic Medicine, UCL Great Ormond Street Institute of Child Health, London, United Kingdom; ^5^ West Midlands Regional Genetics Service, Birmingham Women’s and Children’s NHS Foundation Trust, Birmingham, United Kingdom

**Keywords:** prenatal, exome sequencing, structural defects, genetic diagnosis, implementation

## Abstract

**Introduction:**

Prenatal exome sequencing (pES) can enhance genetic diagnosis of fetuses with structural anomalies and has recently been introduced as a national service in England. We aimed to examine service outcomes such as diagnostic yield (definite final diagnosis), referral rate, and sources of referral, and explore variation in outcomes of pES by individual or service level factors between 01 October 2021 and 30 June 2022.

**Methods:**

pES testing results from the National Health Service laboratories performing testing were linked to National Congenital Anomaly and Rare Disease Registration Service data and the Maternity Services Data Set and descriptive statistics computed.

**Results:**

There were 475,089 women who gave birth in England during the study period. The referral rate for pES was 8.6 (95% CI 7.8, 9.4) per 10,000 maternities. About 59% of those referred proceeded with pES testing and 35% of women who proceeded received a definite final diagnosis with a median turnaround time of 15 days. Of those who had pES testing, 64.6% had a live birth, 25.3% underwent termination of pregnancy (median gestational age at termination: 26 weeks), and 9.3% had a stillbirth. Among the 85 women who had a definite final diagnosis, 40% had a termination of pregnancy, 18% had a stillbirth, and 42% had a live birth. The corresponding figures among women without a definite final diagnosis were 18%, 5%, and 78%, respectively. Among women who had a termination of pregnancy, the median gestational age at final report was 24.9 weeks and 26.2 weeks at termination. Variation observed in some of the characteristics and outcomes between regional services were limited by small sample size.

**Conclusion:**

This study showed that of those referred, pES testing provided a diagnosis for one in three pregnancies with a fetal anomaly across England during the study period when other tests had been non-informative. Women who opted for a termination of pregnancy underwent the procedure at relatively late gestations. Earlier referral for pES, streamlining pathways, and faster turnaround times may help results to be available at an earlier gestation to allow families more time to make decisions around continuing or terminating their pregnancy. The variation in service outcomes between regional services needs to be investigated further to understand the reasons for these differences.

## Introduction

When fetal structural anomalies, occurring in around 2% of pregnancies in the United Kingdom (NHS England. NCARDRS and Congenital Anomaly Official Statistics Report, 2020), are detected by ultrasound, routine prenatal tests such as karyotyping, chromosomal microarray, or gene-specific tests can diagnose an underlying genomic cause in around 40% of cases but many changes that can cause genetic conditions remain undiagnosed (Mone et al., 2021). In unselected pregnancies where there is a structural abnormality and normal karyotype and chromosomal microarray, prenatal exome sequencing (pES) has been shown to improve diagnostic rates by 8%−10% (Lord et al., 2019; Petrovski et al., 2019), with yields increasing further if pre-test selection occurs following multi-disciplinary review and selection for cases likely to have a monogenic aetiology (Mellis et al., 2022). Improved genetic diagnosis allows more accurate parental counselling about prognosis, informs decision-making about pregnancy and perinatal management, overcomes the pre- and postnatal ‘diagnostic odyssey’, and allows accurate counselling about recurrence risk for future pregnancies (Mone et al., 2021).

In October 2018, genetic laboratory services across the National Health Service (NHS) in England were reconfigured to establish a national Genomic Medicine Service (GMS) that is centred on seven regional NHS Genomic Laboratory Hubs (GLHs), with the available genomic tests set out in a national Genomic Test Directory. The aim of having a national GMS is to deliver consolidated, high throughput and high-quality genomic testing with equity of access for patients across the NHS (Hill et al., 2022). In 2020 rapid pES was introduced into clinical practice and offered through the GMS. Referrals are made across England through 17 clinical genetics services who each send samples to their “home” GLH who process and send onwards to one of the two “testing” GLHs (NHS North Thames and NHS Central and South) where sequencing, variant interpretation and reporting are performed (Mone et al., 2021). Parents are eligible for pES when a fetus with multiple multisystem major structural or selected other abnormalities identified on fetal imaging are considered likely to have a monogenetic aetiology and results will impact pregnancy or neonatal management (NHS England and NHS Improvement, 2020). Eligibility for pES is determined by a multidisciplinary team (MDT) that includes fetal medicine experts and clinical geneticists. For more details on the eligibility criteria and clinical examples of included and excluded cases please see the rapid prenatal sequencing service guidance (NHS England and NHS Improvement, 2020).

pES was ordered following a negative aneuploidy test (via QF-PCR) and was performed following or in parallel with chromosomal microarray. pES only proceeded to reporting if chromosomal microarray was negative. Trio (fetus and both parents) sequencing was preferred to aid rapid interpretation. Duo sequencing was performed if one parent was unavailable or if assisted conception (sperm or ovum donation) was used. As trios were performed it was possible to determine parental inheritance patterns. Where possible, analysis was done directly on amniocytes or chorionic villi to enable rapid reporting. In cases where there was insufficient material cells were cultured and used for analysis. All cases were managed by a clinical geneticist who managed cases appropriately including family testing where required.

Analysis of exome data is performed using a panel of more than 1,200 genes that have been determined as likely to cause structural abnormalities detected by prenatal imaging (see Genomics England PanelApp for more details) (Genomics England, 2021). A national group initially reviewed all genes for inclusion on the panel which is now reviewed every 6 months with new genes added accordingly (NHS England and NHS Improvement, 2020). Genes are included if pathogenic variants are considered likely to cause structural abnormalities in the fetus or neonate that are amenable to detection by prenatal imaging. Copy number variants are looked for, but as sensitivity and specificity is unknown, findings are confirmed prior to reporting. Variants are classified according to guidelines (Ellard et al., 2020; Richards et al., 2015), and clinically actionable (pathogenic or likely pathogenic) variants related to the scan findings are reported. Variants of uncertain significance (VUS) can be reported if the MDT review determines that additional information during pregnancy or after birth would allow reclassification of the variant to pathogenic. Incidental findings not related to the indication for testing with implications for child or parental health, or future reproductive risks are reported if multidisciplinary discussion deems it appropriate.

It is important that the pES service is evaluated to determine service outcomes across England to identify any regional differences and allow remedial action if required. This is the first time pES has been offered systematically in a nationally funded healthcare system. In addition, as pES is delivered from seven GLHs there is the potential for wide variation in referrals, uptake, and diagnostic rates and how GLHs and clinicians implement pES in clinical practice. Further potential variation may reflect the size and socio-economic factors of the regions served. This study is a component of the National Institute for Health Research and Care (NIHR) funded study, Optimising EXome PREnatal Sequencing Services (EXPRESS) (Hill et al., 2022), which is evaluating the national delivery of pES through the NHS GMS. The objectives of the aspect of the study reported here were to describe the number and characteristics of women giving birth and service outcomes (referral rate, diagnostic yield and sources of referral) in each GLH area and explore individual- or service-related factors associated with variation in outcomes of pES.

## Materials and methods

The study period was 01 October 2021 to 30 June 2022. We utilised four data sources: pES test data from the two testing GLHs (NHS North Thames GLH and NHS Central and South) (source 1) linked with data from the National Congenital Anomaly and Rare Disease Registration Service (NCARDRS) (Broughan et al., 2024) (source 2) and the Maternity Services Data Set (MSDS) (NHS England, 2024) (source 3) from NHS England, and qualitative and survey data from professionals across the seven GLHs that identified clinical care pathway models used within each GLH (source 4). In addition, data on all women who gave birth in England during the study period were obtained from MSDS to describe the number and characteristics of women giving birth in each GLH area annually and as the denominator for the computation of referral rate.

To ensure linkage to the correct pregnancy, the woman’s NHS number was used to link data from NCARDRS and MSDS where the expected delivery date (EDD) of the pregnancy in one dataset was within 90 days of the other. Where there were multiple matches, the record with the smallest difference in EDD was used.

Where information was taken from MSDS, the most recent valid (not null or unknown) value in each field was used with the exception of booking hospital for which the earliest valid value in the pregnancy was taken to reflect the most accurate organisation at the time of the first antenatal visit (booking) and to avoid misclassification of booking at a tertiary centre, following suspicion of a congenital anomaly. Data from MSDS were also used to source demographic information that was not available in the NCARDRS congenital anomaly registration dataset and adding a field available in MSDS that described the presence of complex social factors. These linked data were then linked to data from the two testing GLHs to examine service outcomes and describe individual or service-related factors associated with variation in outcomes of pES.

### Individual–and service-level characteristics

#### Individual–level characteristics

Individual-level characteristics were categorised. Woman’s age in years was categorised into <20, 20-<25, (collapsed into <25 for GLH data) 25-<30, 30-<35, and ≥35. Ethnicity grouped according to United Kingdom census classification was categorised into White, Asian/Asian British, Black/Black British, Mixed, and Other for the analysis among women who gave birth and White and Black, Asian or Other Minority ethnicity (due to small numbers in the categories other than White ethnicity) for the analysis involving GLH data. The Index of Multiple Deprivation (IMD) quintiles were derived using the Lower Layer Super Output Area (LSOA) 2011 as reference and based on postcode of the woman at booking and complex social factors indicator was defined based on NICE guidance (CG110) (composite variable consisting of yes to either alcohol or drug misuse, recent migrant or asylum seeker status, difficulty reading or speaking English, aged under 20, or domestic abuse) (National Institute for Health and Care Excellence, 2010). The other individual-level characteristic included was gestational age at pregnancy outcome in weeks. All these variables were obtained from NCARDRS and where unavailable data were supplemented from MSDS.

#### Service–level characteristics

In another arm of the EXPRESS study (Walton et al., 2024), the local pES pathway was mapped based on data from interviews (n = 63) and surveys (n = 159) with professionals at each of the 17 clinical genetics services and their linked fetal medicine and obstetric teams. The care pathway models obtained from this mapping were used to create the variable for sources of referral (who initiates and leads the process: genetics, fetal medicine/genetics, or fetal medicine), a service-level characteristic. Other service-level characteristics included turnaround time and gestational age at final report. Turnaround time in days was defined as the number of days between receipt of sample at the testing laboratory and the final report being issued. Turnaround time excludes the time taken to collect and transfer samples to the home GLH, cell culture and DNA extraction and transfer of DNA samples from the home GLH to the testing GLH. Gestational age (weeks) at which the final report was issued was calculated from the estimated date of delivery but not calculated where the date of report was after birth.

### Outcomes

From the GLHs we obtained the proportion of pES testing with a definite final diagnosis (henceforth referred to as ‘diagnosis’ only) (yes vs. no): yes, the proportion of women with a definite final diagnosis from pES (confirmation of the genetic cause of the fetal anomalies by identification of the underlying pathogenic or likely pathogenic DNA variant/s) and no, the proportion of women without a definite final diagnosis from pES (no pathogenic variant or relevant VUS reported). Incidental findings not related to the reason for referral were reported in seven cases and included a variety of rare conditions warranting clinical review of the parent(s) or had implications for future pregnancies. In one case an incidental finding was reported because follow-up and monitoring of the child was warranted. Incidental findings are otherwise not included in this analysis. The pregnancy outcomes of pES were termination (at any gestation), miscarriage (fetal loss up to 24 weeks' gestation), stillbirth (fetal loss over 24 weeks’ gestation), or live birth. These outcomes were obtained using a combination of NCARDRS notified data which is multi-source and supplemented with data from MSDS where it was not available from these sources.

### Statistical analysis

The GLHs were numbered from 1 to 7 and categories for some variables were collapsed for confidentiality reasons to minimise risk of potential disclosure of individual GLHs or individuals. The number of women giving birth in the GLH area annually (mapped based on births in referring units and their associated home births), number of women referred for pES and then proceeding with testing were calculated and their characteristics described using counts and percentages. Referral rates per 10,000 maternities with 95% confidence interval were calculated, overall and for each GLH. Among women who proceeded with pES, sources of referrals and diagnostic yield (based on definite final diagnosis–yes/no) overall and by GLH, and individual- and service-related factors for diagnosis were described using counts and percentages for categorical variables and median (interquartile range (IQR)) for continuous variables. The outcomes of pES and individual- and service-level factors associated with variation in these outcomes were also described using counts and percentages for categorical variables and median (IQR) for continuous variables. Significance testing (2-sided, at 5% significance level) was carried out using chi-square or Fisher’s exact test for categorical variables and Kruskal -Wallis test for continuous variables.

Analyses were conducted using Stata v18.0, R 4.4, and DBeaver (for extraction of data from the NCARDRS Congenital Anomaly PostgreSQL database).

## Approvals

Clinical audits for data collection of pregnancy outcomes were registered for North Thames GLH (GOSH: Reference Number: 3,082) and Central and South GLH (Clinical Audit Registration and Management System (CARMS) at Birmingham Women’s Hospital (CARMS-31001).

## Results

There were 475,089 women who gave birth from 01 October 2021 to 30 June 2022 in England. Overall, among women who gave birth 73% were of White ethnicity, 27% were from the most deprived quintile, and 14% had at least one complex social factor. These factors varied by GLH (Table 1) (*p* < 0.001).

**TABLE 1:** Characteristics of women giving birth in each GLH area.

	GLH 1 (n = 56,349)	GLH 2 (n = 112,864)	GLH 3 (n = 54,248)	GLH 4 (n = 32,171)	GLH 5 (n = 86,582)	GLH 6 (n = 68,386)	GLH 7 (n = 64,489)	Total (N = 475,089)	*p*-value
	n (%)	n (%)	n (%)	n (%)	n (%)	n (%)	n (%)	n (%)	
Maternal age, years									
<20	2,086 (3.7)	2,406 (2.1)	1,367 (2.5)	1,062 (3.3)	3,109 (3.6)	3,397 (5.0)	2,374 (3.7)	15,801 (3.3)	<0.001
20-<25	8,311 (14.7)	12,370 (11.0)	5,702 (10.5)	4,126 (12.8)	12,363 (14.3)	11,170 (16.3)	9,301 (14.4)	63,343 (13.3)	
25-<30	16,323 (29.0)	28,121 (24.9)	12,785 (23.6)	8,512 (26.5)	24,639 (28.5)	20,368 (29.8)	18,502 (28.7)	129,250 (27.2)	
30-<35	18,278 (32.4)	39,281 (34.8)	19,198 (35.4)	11,294 (35.1)	28,332 (32.7)	21,021 (30.7)	21,099 (32.7)	158,503 (33.4)	
≥35	11,351 (20.1)	30,686 (27.2)	15,196 (28.0)	7,177 (22.3)	18,139 (21.0)	12,430 (18.2)	13,213 (20.5)	108,192 (22.8)	
Maternal Ethnicity									
White	43,551 (80.8)	65,430 (59.3)	37,264 (70.8)	28,033 (89.8)	58,126 (71.4)	53,728 (79.7)	46,945 (75.8)	33,3077 (72.6)	<0.001
Asian/Asian British	5,745 (10.7)	24,273 (22.0)	5,139 (9.8)	1,101 (3.5)	13,382 (16.4)	7,618 (11.3)	8,599 (13.9)	65,857 (14.4)	
Black/Black British	2,058 (3.8)	9,174 (8.3)	5,512 (10.5)	762 (2.4)	4,815 (5.9)	2,348 (3.5)	2,594 (4.2)	27,263 (5.9)	
Mixed	1,191 (2.2)	3,533 (3.2)	1,916 (3.6)	596 (1.9)	2,205 (2.7)	1,275 (1.9)	1,233 (2.0)	11,949 (2.6)	
Other	1,377 (2.6)	7,933 (7.2)	2,774 (5.3)	715 (2.3)	2,842 (3.5)	2,470 (3.7)	2,528 (4.1)	20,639 (4.5)	
*Missing*	*2,427*	*2,521*	*1,643*	*964*	*5,212*	*947*	*2,590*	*16,304*	
Index of Multiple Deprivation									
Quintile 1 (Most deprived)	12,160 (22.0)	20,520 (18.3)	10,175 (18.8)	4,945 (15.4)	24,426 (28.9)	25,588 (37.6)	27,023 (42.5)	124,837 (26.6)	<0.001
Quintile 2	11,008 (19.9)	31,682 (28.2)	14,288 (26.4)	7,191 (22.4)	14,949 (17.7)	13,942 (20.5)	12,089 (19.0)	105,149 (22.2)	
Quintile 3	11,203 (20.3)	23,670 (21.1)	12,069 (22.3)	8,153 (25.4)	15,027 (17.8)	10,288 (15.1)	9,072 (14.3)	89,482 (19.1)	
Quintile 4	10,924 (19.8)	18,833 (16.8)	9,667 (17.9)	6,777 (21.1)	15,197 (18.0)	10,226 (15.0)	8,353 (13.1)	79,977 (17.1)	
Quintile 5 (Least deprived)	9,988 (18.1)	17,666 (15.7)	7,823 (14.5)	5,014 (15.6)	14,897 (17.6)	7,974 (11.7)	7,075 (11.1)	70,437 (15.0)	
*Missing*	*1,066*	*493*	*226*	*91*	*2,086*	*368*	*877*	*5,207*	
Complex social factor indicator									
No	49,913 (88.6)	94,485 (85.0)	47,293 (87.2)	28,834 (90.1)	71,121 (85.5)	58,602 (86.8)	53,470 (84.5)	403,718 (86.3)	<0.001
Yes	6,393 (11.4)	16,679 (15.0)	6,943 (12.8)	3,177 (9.9)	12,091 (14.5)	8,874 (13.2)	9,801 (15.5)	63,958 (13.7)	
*Missing*	*43*	*1,700*	*12*	*160*	*3,370*	*910*	*1,218*	*7,413*	

Note: *p*-values from chi-square test.

Abbreviation: GLH: genomic laboratory hub.

**FIGURE 1:**
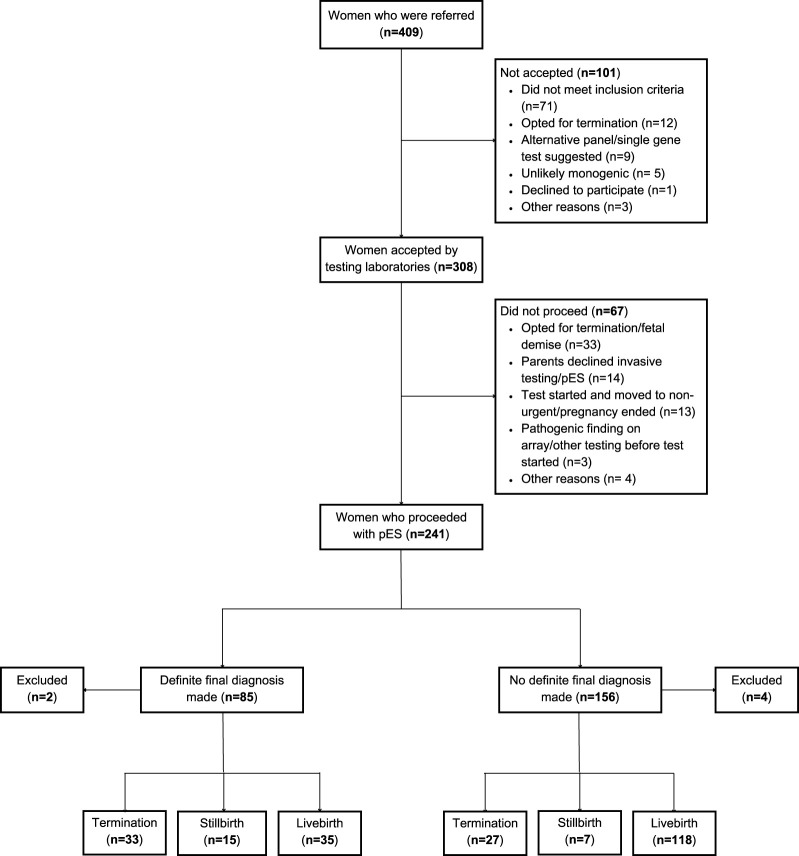
Flowchart of women in the EXPRESS study. Note: The total for pregnancy outcomes is 235 (Definite final diagnosis: 83 and No definite final diagnosis: 152) instead of 241 because 6 women were excluded from the analysis (miscarriage: 2 and 4: missing data for pregnancy outcome).

In total, 409 women were referred for pES equating to a referral rate of 8.6 (95% CI 7.8, 9.4) per 10,000 maternities. The rates ranged from 4.3 for GLH 2 to 11.9 for GLH 1. Of those referred, 75.3% (308/409) were accepted by the testing laboratories and 58.9% (241/409) pES tests proceeded (Table 2). The individual reasons for the test not being accepted by the testing laboratory were not recorded, but included that the abnormalities did not meet eligibility criteria, the parents decided to terminate the pregnancy or declined invasive testing, or that some other test was deemed more appropriate. The reason for not proceeding with pES test was available for 66 women–the primary reason being pregnancy ending due to fetal demise or termination (n = 33) followed by parents declining invasive testing or pES (n = 14) (Figure 1). The overall characteristics of women who were referred or who proceeded did not differ substantially from the population of women who gave birth in the GLH areas. The majority of women who were referred or who proceeded with pES were more than 30 years of age and of White ethnicity with wide variations between GLHs. There was evidence of a dose-response relationship with IMD: a lower proportion of women referred (*p* < 0.001) or proceeded (*p* = 0.002) with pES were from the least deprived areas and a higher proportion were from the most deprived areas. However, comparisons at the GLH-level should be interpreted with caution due to low numbers (Tables 3 and 4).

**TABLE 2:** Characteristics of women who were referred for prenatal ES.

	Accepted by testing laboratory (n = 308)^a^		Not accepted by GLH (n = 101) n (%)	Total (N = 409) n (%)
	Proceeded with prenatal ES (n = 241) n (%)	Not proceeded with prenatal ES (n = 67) n (%)		
Maternal age, years				
<25	35 (14.5)	4 (6.3)	11 (12.6)	50 (12.8)
25-<30	61 (25.3)	20 (31.2)	16 (18.4)	97 (24.7)
30-<35	85 (35.3)	19 (29.7)	27 (31.0)	131 (33.4)
≥35	60 (24.9)	21 (32.8)	33 (37.9)	114 (29.1)
*Missing*	*0*	*3*	*14*	*17*
Maternal Ethnicity				
White	153 (74.6)	14 (87.5)	14 (77.8)	181 (75.7)
Black, Asian, and Other Minority	52 (25.4)	2 (12.4)	4 (22.2)	58 (24.3)
*Missing*	36	*51*	*83*	*170*
Index of Multiple Deprivation				
Quintile 1 (Most deprived)	54 (23.8)	14 (26.4)	19 (25.7)	87 (24.6)
Quintile 2	48 (21.2)	9 (17.0)	16 (21.6)	73 (20.6)
Quintile 3	47 (20.7)	8 (15.1)	18 (24.3)	73 (20.6)
Quintile 4	41 (18.1)	12 (22.6)	11 (14.9)	64 (18.1)
Quintile 5 (Least deprived)	37 (16.3)	10 (18.9)	10 (13.5)	57 (16.1)
*Missing*	*14*	*14*	*27*	*55*

^a^ 413 women were referred but 4 women requested to withdraw before approval decision.

*p*-values for comparison of characteristics for accepted vs. not accepted and for proceeded vs. no proceeded are greater than 0.05.

n (%) for women who proceeded with pES are different from Tables 4, 6, and 8 because data used to construct this table were obtained from GLHs alone as referral data were not available in the linked data due to data restrictions.

Abbreviations: ES: exome sequencing, GLH: genomic laboratory hub.

**TABLE 3:** Characteristics of women who were referred for prenatal ES in each GLH.

	GLH 1 (n = 67)	GLH 2 (n = 49)	GLH 3 (n = 73)	GLH 4 (n = 23)	GLH 5 (n = 88)	GLH 6 (n = 49)	GLH 7 (n = 60)	Total (N = 409)	*p*-value
	n (%)	n (%)	n (%)	n (%)	n (%)	n (%)	n (%)	n (%)	
Referral rate per 10,000 maternities	11.9	4.3	13.5	7.1	10.2	7.2	9.3	8.6	
Maternal age, years									
<25	9 (14.1)	4 (8.2)	6 (8.3)	1 (4.5)	10 (12.0)	8 (17.4)	12 (21.4)	50 (12.8)	0.35
25-<30	16 (25.0)	9 (18.4)	20 (27.8)	3 (13.6)	21 (25.3)	15 (32.6)	13 (23.2)	97 (24.7)	
30-<35	21 (32.8)	22 (44.9)	25 (34.7)	9 (40.9)	29 (34.9)	14 (30.4)	11 (19.6)	131 (33.4)	
≥35	18 (28.1)	14 (28.6)	21 (29.2)	9 (40.9)	23 (27.7)	9 (19.6)	20 (35.7)	114 (29.1)	
*Missing*	*3*	*0*	*1*	*1*	*5*	*3*	*4*	*17*	
Maternal Ethnicity									
White	29 (78.4)	16 (61.5)	34 (68.0)	6 (100.0)	49 (77.8)	14 (60.9)	33 (97.1)	181 (75.7)	0.007
Black, Asian, and Other Minority	8 (21.6)	10 (38.5)	16 (32.0)	0 (0.0)	14 (22.2)	9 (39.1)	1 (2.9)	58 (24.3)	
*Missing*	*30*	*23*	*23*	*17*	*25*	*26*	*26*	*170*	
Index of Multiple Deprivation									
Quintile 1 (Most deprived)	16 (26.2)	13 (27.7)	4 (6.0)	1 (5.0)	14 (20.3)	20 (51.3)	19 (37.3)	87 (24.6)	<0.001
Quintile 2	11 (18.0)	16 (34.0)	16 (23.9)	3 (15.0)	13 (18.8)	6 (15.4)	8 (15.7)	73 (20.6)	
Quintile 3	9 (14.8)	9 (19.1)	23 (34.3)	6 (30.0)	13 (18.8)	4 (10.3)	9 (17.6)	73 (20.6)	
Quintile 4	16 (26.2)	4 (8.5)	12 (17.9)	6 (30.0)	12 (17.4)	6 (15.4)	8 (15.7)	64 (18.1)	
Quintile 5 (Least deprived)	9 (14.8)	5 (10.6)	12 (17.9)	4 (20.0)	17 (24.6)	3 (7.7)	7 (13.7)	57 (16.1)	
*Missing*	*6*	*2*	*6*	*3*	*19*	*10*	*9*	*55*	

Note: Complex social factor variable not included because it was available only for women who proceeded with prenatal ES.

*p*-values from chi-square test.

Abbreviations: ES: exome sequencing, GLH: genomic laboratory hub.

**TABLE 4:** Characteristics of women who proceeded with prenatal ES in each GLH.

	GLH 1 (n = 36)	GLH 2 (n = 22)	GLH 3 (n = 48)	GLH 4 (n = 11)	GLH 5 n = 63)	GLH 6 (n = 28)	GLH 7 (n = 33)	Total (N = 241)	*p*-value
	n (%)	n (%)	n (%)	n (%)	n (%)	n (%)	n (%)	n (%)	
Maternal age, years									
<25	3 (8.3)	3 (13.6)	4 (8.3)	1 (9.1)	11 (17.5)	4 (14.3)	8 (24.2)	34 (14.1)	0.26
25-<30	9 (25.0)	5 (22.7)	10 (20.8)	1 (9.1)	14 (22.2)	10 (35.7)	8 (24.2)	57 (23.7)	
30-<35	15 (41.7)	8 (36.4)	21 (43.8)	7 (63.6)	18 (28.6)	10 (35.7)	4 (12.1)	83 (34.4)	
≥35	9 (25.0)	6 (27.3)	13 (27.1)	2 (18.2)	20 (31.7)	4 (14.3)	13 (39.4)	67 (27.8)	
Maternal Ethnicity									
White	23 (79.3)	8 (42.1)	36 (76.6)	11 (100.0)	47 (81.0)	18 (64.3)	25 (89.3)	168 (76.4)	0.002
Black, Asian, and Other Minority	6 (20.7)	11 (57.9)	11 (23.4)	0 (0.0)	11 (19.0)	10 (35.7)	3 (10.7)	52 (23.6)	
*Missing*	*7*	*3*	*1*	*0*	*5*	*0*	*5*	*21*	
Index of Multiple Deprivation									
Quintile 1 (Most deprived)	11 (32.4)	6 (31.6)	1 (2.1)	0 (0.0)	16 (25.8)	14 (50.0)	9 (29.0)	57 (24.6)	0.002
Quintile 2	6 (17.6)	8 (42.1)	13 (27.7)	2 (18.2)	10 (16.1)	8 (28.6)	6 (19.4)	53 (22.8)	
Quintile 3	4 (11.8)	3 (15.8)	15 (31.9)	3 (27.3)	9 (14.5)	3 (10.7)	8 (25.8)	45 (19.4)	
Quintile 4	8 (23.5)	1 (5.3)	10 (21.3)	3 (27.3)	14 (22.6)	2 (7.1)	6 (19.4)	44 (19.4)	
Quintile 5 (Least deprived)	5 (14.7)	1 (5.3)	8 (17.0)	3 (27.3)	13 (21.0)	1 (3.6)	2 (6.5)	33 (14.2)	
*Missing*	*2*	*3*	*1*	*0*	*1*	*0*	*2*	*9*	
Complex social factor indicator									
No	26 (86.7)	18 (90.0)	44 (93.6)	10 (90.9)	56 (91.8)	22 (78.6)	25 (86.2)	201 (88.9)	0.53
Yes	4 (13.3)	2 (10.0)	3 (6.4)	1 (9.1)	5 (8.2)	6 (21.4)	4 (13.8)	25 (11.1)	
*Missing*	*6*	*2*	*1*	*0*	*2*	*0*	*4*	*15*	

Note: *p*-values from chi-square test except for complex social factor for which Fisher’s exact test was used.

Abbreviations: ES: exome sequencing, GLH: genomic laboratory hub.

One-third of women who underwent pES received a diagnosis, with variation across GLHs ranging from 28.6% to 45.5% (Table 5). The median turnaround time was 16 days when a diagnosis was made and 14 days when there was no diagnosis (Table 6). There were differences in ethnicity (*p* = 0.01), complex social factor indicator (*p* = 0.02), and gestational age at pregnancy outcome (*p* = 0.001) for women with a diagnosis compared to those without a diagnosis (Table 6). Among women who had a diagnosis, 67% were of White ethnicity and 18% had a complex social factor compared to 82% and 7.5%, respectively, among women who did not have a diagnosis (Table 6).

**TABLE 5:** Service outcomes (definite final diagnosis and sources of referral) among women who proceeded with prenatal ES in each GLH.

	GLH 1 (n = 36)	GLH 2 (n = 22)	GLH 3 (n = 48)	GLH 4 (n = 11)	GLH 5 n = 63)	GLH 6 (n = 28)	GLH 7 (n = 33)	Total (N = 241)	*p*-value
	n (%)	n (%)	n (%)	n (%)	n (%)	n (%)	n (%)	n (%)	
Definite diagnosis (Diagnostic yield)									
No	21 (58.3)	15 (68.2)	30 (62.5)	6 (54.5)	43 (68.3)	20 (71.4)	21 (63.6)	156 (64.7)	0.89
Yes	15 (41.7)	7 (31.8)	18 (37.5)	5 (45.5)	20 (31.7)	8 (28.6)	12 (36.4)	85 (35.3)	
Sources of Referral									
Genetics	1 (2.9)	11 (50.0)	48 (100.0)	11 (100.0)	26 (52.0)	20 (71.4)	9 (27.3)	126 (55.5)	NC
Fetal medicine/Genetics	25 (71.4)	11 (50.0)	0 (0.0)	0 (0.0)	24 (48.0)	7 (25.0)	24 (72.7)	91 (40.1)	
Fetal medicine	9 (25.7)	0 (0.0)	0 (0.0)	0 (0.0)	0 (0.0)	1 (3.6)	0 (0.0)	10 (4.4)	
*Missing*	*1*	*0*	*0*	*0*	*13*	*0*	*0*	*14*	

Note: Diagnostic yield: Yes: Pathogenic variant reported, No: VUS, or no pathogenic variant reported.

*p*-value from chi-square test.

Abbreviations: ES: exome sequencing, GLH: genomic laboratory hub, NC: Not computed (because of multiple zeroes for fetal medicine).

**TABLE 6:** Individual and service-related factors according to definite final diagnosis among women who proceeded with prenatal ES.

	Diagnosis made (n = 85)	No diagnoses made (n = 156)	Total (N = 241)	*p*-value
	n (%)	n (%)	n (%)	
Individual-level				
Maternal age, years				
<25	13 (15.3)	21 (13.5)	34 (14.1)	0.52
25-<30	23 (27.1)	34 (21.8)	57 (23.6)	
30-<35	25 (29.4)	58 (37.2)	83 (34.4)	
≥35	24 (28.2)	43 (27.6)	67 (27.8)	
Maternal Ethnicity				
White	52 (66.7)	116 (81.7)	168 (76.4)	0.01
Black, Asian, and Other Minority	26 (33.3)	26 (18.3)	52 (23.6)	
*Missing*	*7*	*14*	*21*	
Index of Multiple Deprivation				
Quintile 1 (Most deprived)	23 (28.4)	34 (22.5)	57 (24.6)	0.25
Quintile 2	21 (25.9)	32 (21.2)	53 (22.8)	
Quintile 3	16 (19.8)	29 (19.2)	45 (19.4)	
Quintile 4	9 (11.1)	35 (23.2)	44 (19.0)	
Quintile 5 (Least deprived)	12 (14.8)	21 (13.9)	33 (14.2)	
*Missing*	*4*	*5*	*9*	
Complex social factor indicator				
No	65 (82.3)	136 (92.5)	201 (88.9)	0.02
Yes	14 (17.7)	11 (7.5)	25 (11.1)	
*Missing*	6	9	15	
Gestational age at outcome, weeks, median (IQR)	32.9 (27.1–37.8)	37.0 (33.8–38.7)	36.6 (30.9–38.4)	0.001
*Missing*	*15*	*10*	*25*	
Service-level				
Sources of Referral				
Genetics	46 (57.5)	80 (54.4)	126 (55.5)	0.82
Fetal medicine/Genetics	30 (37.5)	61 (41.5)	91 (40.1)	
Fetal medicine	4 (5.0)	6 (4.1)	10 (4.4)	
*Missing*	*5*	*9*	*14*	
Turnaround time, days, median (IQR)	16 (14–21)	14 (12.8–17)	15 (13–20)	<0.001
Gestational age at final report, weeks, median (IQR)	26.2 (23.9–29.5)	26.6 (24.3–31.0)	26.4 (24.1–30.3)	0.46
*Missing*	*1*	*0*	*1*	

Note: Diagnostic yield: Yes: Pathogenic variant reported, No: VUS, or no pathogenic.

variant reported.

*p*-values from chi-square test except for continuous outcomes for which the Kruskal Wallis test was used.

Abbreviation: IQR: Inter-quartile range, ES: exome sequencing.

Sixty-five percent of women who had pES had live births, 9% had a stillbirth and 25.3% of women underwent termination (Table 7). About 1/3rd of women with complex social factors underwent termination of pregnancy. There were differences observed in pregnancy outcomes between those with and without a diagnosis (*p* < 0.001) – among women who had a diagnosis 39.8% underwent termination of pregnancy, 18.1% had a stillbirth, and 42.2% had a live birth whereas among women without a diagnosis, 17.8% underwent termination of pregnancy, 4.6% had a stillbirth, and 77.6% had a live birth (Table 8). Among women who had a termination of pregnancy, the median gestational age at final report was 24.9 weeks and at termination it was 26.2 weeks.

**TABLE 7:** Pregnancy outcomes among women who proceeded with prenatal ES.

	GLH 1 (n = 36)	GLH 2 (n = 22)	GLH 3 (n = 48)	GLH 4 (n = 11)	GLH 5 (n = 63)	GLH 6 (n = 28)	GLH 7 (n = 33)	Total (N = 241)
	n (%)	n (%)	n (%)	n (%)	n (%)	n (%)	n (%)	n (%)
Termination	7 (20.0)	3 (14.3)	18 (38.3)	3 (27.3)	20 (32.3)	3 (11.5)	6 (18.2)	60 (25.3)
Stillbirth	5 (14.3)	3 (14.3)	1 (2.1)	2 (18.2)	4 (6.5)	3 (11.5)	4 (12.1)	22 (9.3)
Livebirth	23 (65.7)	15 (71.4)	28 (59.6)	6 (54.5)	38 (61.3)	20 (76.9)	23 (69.7)	153 (64.6)
*Miscarriage/Missing*	*1*	*1*	*1*	*0*	*1*	*2*	*0*	*6*

Abbreviations: ES: exome sequencing, GLH: genomic laboratory hub.

Note: *p*-value for comparison of pregnancy outcomes by GLH, 0.21.

**TABLE 8:** Individual and service-related factors for pregnancy outcomes among women who proceeded with prenatal ES.

	Termination (n = 60)	Stillbirth (n = 22)	Livebirth (n = 153)	Total (N = 235)	*p*-value
	n (%)	n (%)	n (%)	n (%)	
<25	4 (11.8)	4 (11.8)	26 (76.5)	34 (14.5)	0.28
25-<30	15 (26.8)	7 (12.5)	34 (60.7)	56 (23.8)	
30-<35	20 (25.3)	4 (5.1)	55 (69.6)	79 (33.6)	
≥35	21 (31.8)	7 (10.6)	38 (57.6)	66 (28.1)	
Maternal Ethnicity					
White	46 (28.0)	13 (7.9)	105 (64.0)	164 (76.3)	0.29
Black, Asian, and Other Minority	10 (19.6)	7 (13.7)	34 (66.7)	51 (23.7)	
*Missing*	*4*	*2*	*14*	*20*	
Index of Multiple Deprivation					
Quintile 1 (Most deprived)	7 (12.3)	4 (7.0)	46 (80.7)	57 (25.1)	0.08
Quintile 2	15 (31.3)	8 (16.7)	25 (52.1)	48 (21.1)	
Quintile 3	12 (26.7)	3 (6.7)	30 (66.7)	45 (19.8)	
Quintile 4	13 (29.5)	5 (11.4)	26 (59.1)	44 (19.5)	
Quintile 5 (Least deprived)	12 (36.4)	2 (6.1)	19 (57.6)	33 (14.5)	
*Missing*	*1*	*0*	*7*	*8*	
Complex social factor indicator					
No	50 (25.5)	18 (9.2)	128 (65.3)	196 (88.7)	0.66
Yes	8 (32.0)	3 (12.0)	14 (56.0)	25 (11.3)	
*Missing*	*2*	*1*	*11*	*14*	
Turnaround time, days, median (IQR)	16 (13–21)	15 (13–25.5)	14 (13.0–19.0)	15 (13–20)	0.26
*Missing*	0	0	0	2	
Gestational age at final report, weeks, median (IQR)	24.9 (22.1–27.3)	27.3 (25.5–29.9)	27.1 (24.7–31.8)	26.5 (24.3–30.4)	0.001
*Missing*	1	0	0	3	
Gestational age at outcome, weeks, median (IQR)	26.2 (23.5–31.1)	30.6 (27.8–32.7)	37.9 (36.0–38.9)	36.4 (30.9–39.4)	<0.001
*Missing*	14	0	7	23	
Definite diagnosis (Diagnostic yield)					
No	27 (17.8)	7 (4.6)	118 (77.6)	152 (64.7)	<0.001
Yes	33 (39.8)	15 (18.1)	35 (42.2)	83 (35.3)	

Note: Row percentages presented except column percentage for total.

The total is 235 instead of 241 because 6 women were excluded from these analyses (miscarriage: 2 and missing data for pregnancy outcome: 4).

*p*-values from chi-square test except for continuous outcomes for which the Kruskal Wallis test was used.

Abbreviations: ES: exome sequencing.

No material differences were observed by sources of referral (who initiates and leads the process–fetal medicine, genetics or fetal medicine and genetics) that were identified across the 17 genetics services in England (Supplementary Table S1).

## Discussion

This study showed that in the new NHS GMS in England the referral rate for pES was 8.6 (95% CI 7.8, 9.4) per 10,000 maternities during 01 October 2021 to 30 June 2022. More than half of the women who were referred for pES had the test and 1 in 3 women who accepted testing received a definite final diagnosis. At GLH level, there were differences in the characteristics of women who gave birth especially by ethnicity and IMD. There were differences in women’s ethnicity, complex social factor indicator, and gestational age at pregnancy outcome by type of result–diagnosis made or ‘no informative’ result. Pregnancy outcomes varied by ethnicity, IMD, and complex social factor. There was variation in some of the characteristics and outcomes between GLHs but the differences must be interpreted with caution due to the low sample size at GLH level.

A higher proportion of women who were referred for pES were over 30 years of age, White, and from the most deprived areas. However, women who gave birth shared similar characteristics to those who were referred. This may indicate that referral rates could be dependent on factors other than the selected characteristics included in the study.

Ethnicity and complex social factors were important factors for confirmed diagnosis and pregnancy outcomes, with the proportion of women from an Asian background or with complex social factors higher in the group with a diagnosis, and a higher proportion of women with complex social factors undergoing termination of pregnancy. The higher proportion of women from an Asian background in the group with a diagnosis may, at least in part, reflect the increased occurrence of recessive genetic disorders in communities with a South Asian background where consanguineous marriages are more common (Corry, 2014; Small et al., 2017). However, we were unable to examine this in our study due to lack of data on consanguinity and autosomal recessive diagnoses caused by homozygous identical DNA variants. The higher proportion of women from an Asian background in the diagnostic group may be linked to the higher proportion of women in this group with complex social factors as the variable, complex social factor, includes recent migrants (National Institute for Health and Care Excellence, 2010).

A systematic review by Mellis et al. reported a wide range for diagnostic yield from pES (5%–89%) and a pooled incremental diagnostic yield of 31% with high heterogeneity between the 66 included studies (Mellis et al., 2022). The diagnostic yield (based on definite final diagnosis) in our study of 35% is within this range and aligns with the higher diagnostic yields seen in the Mellis et al. review when cases were pre-selected for likelihood of a monogenic condition compared to studies where cases were unselected (45% vs. 15%) (Mellis et al., 2022). This finding is expected because a key component of the eligibility criteria for the English pES service is that the fetus is considered likely to have a monogenic aetiology following MDT review. The proportion of women with a diagnosis did vary between GLHs, ranging from 29% to 46%, but not statistically significant possibly due to low numbers at GLH level. The variation between GLHs was not explained by differences in who leads the local services (genetics, fetal medicine or genetics, and fetal medicine) as no material difference in diagnostic yield was found. In the literature, common reasons reported for differences in diagnostic yield include differences in inclusion criteria, use of trio (parents and fetus) *versus* fetus only sequencing, inclusion of fetuses with multiple anomalies (Petrovski et al., 2019) or in cases preselected following genetic review (Lord et al., 2019; Petrovski et al., 2019; Mellis et al., 2022). As most of these factors are fixed across the national pES service, the variation in diagnosis seen between GLHs is more likely to be due to differences in demographics of the regional population, and how the eligibility criteria are applied locally and by the two testing laboratories. Qualitative interviews of professionals involved in delivery of pES that is reported elsewhere showed potential variation in referrals from local obstetric units, as some described difficulties engaging peripheral maternity units (Peter et al., 2024). To ensure equity of access for parents, further education for healthcare professionals and review of local process are needed to ensure the eligibility criteria are applied in a similar way across all regions.

Having a diagnosis seems to have influenced the decision to terminate or proceed with pregnancy as evidenced by the higher proportion of termination of pregnancy among women who had a diagnosis compared to women without a diagnosis (40% vs. 18%). This may reflect the fact that having a definitive genetic diagnosis allows for better prediction of prognosis, which may often include features that cannot be determined from a fetal scan, such as developmental delay or other factors that may influence quality of life. Whilst not having identified a genetic aetiology may be reassuring to some extent and parents prefer to live with the uncertainty. A similar impact of pES findings on decision making around termination of pregnancy has been seen in other studies considering pregnancy outcomes following pES, when offered in the NHS GMS at a single centre (Poljak et al., 2023) as well as in two studies from the Netherlands (Deden et al., 2020). Moreover, in qualitative research, parents who had pES have described how they valued the information from pES results for decision-making around whether or not to terminate pregnancy (McInnes et al., 2024). Notably in this study, 42% of women with a diagnosis continued the pregnancy and had a live birth, highlighting that findings from pES also lead to decisions to continue the pregnancy and can be used to inform pregnancy management and neonatal care (Mellis et al., 2022; McInnes et al., 2024). As the eligibility criteria for pES tests will continue to evolve over time and may become much broader it will be important to conduct future research that continues to examine the reasons parents take up testing and how pES results influence their decisions about continuing or terminating the pregnancy.

It is important to note that the median age for termination of pregnancy was 26.2 weeks. This timeframe aligns with the fact that the majority of referrals for pES are made following the routine fetal anomaly scan at 18–20 weeks, which, when combined with the time required for pre-test counselling, sample transfer, laboratory testing, returning results and decision making, frequently pushes termination options to later in pregnancy. Furthermore, some anomalies develop during pregnancy, in particular, those associated with brain malformations or movement disorders which may then not present until later in pregnancy. Notably, the proportion of terminations performed for fetal anomalies after 24 weeks has not changed since the introduction of the pES service (0.13% in 2019, 0.11% in 2020 and 0.10% in 2022) (Poljak et al., 2023; Department of Health and Social Care, 2019; Department of Health and Social Care, 2020; Office for Health Improvement and Disparities, 2022). In England the legal basis for termination of pregnancy changes at 24 weeks gestation and is only permitted if “there is a substantial risk that if the child were born it would suffer from such physical or mental abnormality as to be seriously handicapped”. Accordingly, both parents and professionals describe the impacts on parents of an anxious wait for pES results when timelines approach 24 weeks (Mellis et al., 2022; McInnes et al., 2024). Turnaround time for testing is also crucial for getting results to families earlier in pregnancy and options for streamlining pathways should be considered.

In this study, we observed that the turnaround time between DNA samples arriving at the laboratory and issuing the final report was 16 days for a diagnosis result and 14 days when there was no diagnosis made. Turnaround times are shorter than reported in other studies, with Mellis et al. reporting a median turnaround time of 20 days in their systematic review of 66 studies of pES conducted in research and clinical settings (Mellis et al., 2022). In addition, a previous study looking at the English pES service conducted in a single fetal medicine unit in England also reported a longer turnaround time for cases with a confirmed diagnosis (22 days vs. 14 days) (Poljak et al., 2023). Possible reasons for the difference in turnaround time between diagnosis and no diagnosis results are the necessity for validation of some pathogenic/likely pathogenic variants before reporting (Poljak et al., 2023; Normand et al., 2018). In addition, the challenges of variant interpretation requires close communication between the laboratory and referring clinicians. Results may also be delayed if the fetal phenotype evolves, or if sequencing findings require additional examination of the fetus or parents (Chandler et al., 2022). Turnaround time is critical because parents and clinicians will use the test results for decision-making including termination, pregnancy management, delivery planning, and neonatal treatment. A shorter turnaround time will allow more time for parents and clinicians to make decisions about next steps. In addition to the turnaround time at the testing laboratory, local service factors are also crucial for the speed with which results are returned to parents, for example, local pathways could be streamlined to reduce the time taken for samples to reach the testing laboratories and local audits of consent processes, sample collection and transfer may help to identify areas for improvement.

This is the first study to examine potential variation in individual or service level factors related to referrals to the NHS GMS pES service and service outcomes. A limitation here is that we could not explore differences at the GLH or individual service-level in more detail due to the relatively small sample size. Another limitation is missing values especially for data collected by the testing GLHs with highest proportion for ethnicity (8.7%) and gestational age at outcome (10.4%). We could not assess differences between GLHs for diagnosis after accounting for potential confounders due to limited sample size at GLH-level. It was outside the scope of this work to look at the pregnancy outcomes where cases were referred but did not proceed to pES as they did not meet the eligibility criteria or the pregnancy ended, this should be considered in future research. Another limitation is that, we did not have data for non-referred cases because of which we could not compare outcomes between referred and non-referred cases. It should also be noted that we have studied at a 9 month window of a newly implemented service which continues to evolve over time. This may be particularly crucial as the service was launched in the middle of the COVID pandemic when clinical practices changed significantly. Finally, our study was conducted in a national healthcare setting and some of the issues reflect the laws around termination of pregnancy in England, as such there are limitations on the generalisability of our findings for other settings.

In summary, we observed that the characteristics of women having pES mirrored those of women giving birth across England. Differences were observed between GLHs in service outcomes of pES. There were also differences in characteristics observed for pregnancy outcomes that may guide clinicians to provide additional support for certain groups of women when making decisions around pES and management of pregnancy and birth. pES is guiding decisions around whether to continue or end the pregnancy as evidenced by the fact that, pregnancy terminations occurred relatively late and were more common in women who had a diagnosis than women without a diagnosis. Future research needs to focus on how we might improve the pES service to enable earlier referral for pES and faster turnaround times to allow more time for women and families to make informed decisions, whenever possible.

## EXPRESS Clinical Outcomes Group

Ruth Armstrong, Tazeen Ashraf, Ana Beleza-Meireles, Marta Bertoli, Lucy Bownass, Jennifer Campbell, Natalie Canham, Ruth Cleaver, Jan Cobben, Jacqueline Eason, Nour Elkhateeb, Alice Gardham, Alice Garrett, Sara Hillman, Emma Hobson, Simon Holden, Muriel Holder-Espinasse, Tessa Homfray, Monika Kosicka-Slawinska, Alison Male, Sahar Mansour, Sarju G. Mehta, Cathryn Moss, Jessica Myring, Pranav Pandya, Katrina Prescott, Lorna Randall, Sarah Richardson, Alexander Ross, Alison Stewart, Dagmar Tapon, Hannah Titheradge, Pradeep Vasudevan, Astrid Weber, Louise Wilson.

## Data Availability

The datasets presented in this article are not readily available because the data cannot be shared because of confidentiality issues and to comply with the United Kingdom GDPR regulations. Data cannot be accessed by researchers external to the project.
