# Supplementary material for: Implementation of a national rapid prenatal exome sequencing service in England: evaluation of service outcomes and factors associated with regional variation
Source: Front Genet. 2024 Nov 6;15:1485306. doi: 10.3389/fgene.2024.1485306 (PMC11576421; doi:10.3389/fgene.2024.1485306)
Supplement: Supplementary file 1 [file Table1.docx]

**Supplementary Table.** EXPRESS Model Care Pathways

| **Model** | **Who initiates and leads the process  (defined as who takes consent)** | **Which staff are involved in service (core staffing throughout)** | **Sites that fit this model**  **Genetics service (GLH)** |
| --- | --- | --- | --- |
| Model 1 | Genetics | Fetal medicine consultant  Clinical geneticist  Clinical scientist | **St George’s (South East)** |
| Model 2 | Fetal medicine or Genetics | Fetal medicine consultant  Clinical geneticist  Clinical scientist | **Newcastle (North East & Yorkshire)** |
| Model 3 | Fetal medicine | Fetal medicine consultant  Clinical geneticist  Clinical scientist  Genetic counsellor | **Sheffield (North East & Yorkshire)**  **Nottingham (East)** |
| Model 4 | Genetics  [Fetal medicine rarely or only recently] | Fetal medicine consultant  Clinical geneticist  Clinical scientist  Genetic counsellor | **Leeds (North East & Yorkshire)**  **Bristol (South West)**  **Exeter (South West)**  **Manchester (North West)**  **Oxford (Central and South)** |
| Model 5 | Fetal Medicine or Genetics | Fetal medicine consultant  Clinical geneticist  Clinical scientist  Midwife | **Liverpool (North West)**  **Cambridge (East)** |
| Model 6 | Genetics  [Fetal medicine rarely or only recently] | Fetal medicine consultant  Clinical geneticist  Clinical scientist | **Wessex (Central and South)**  **GOSH (North Thames)**  **Guys and St Thomas (South East)** |
| Model 7 | Fetal medicine or Genetics | Fetal medicine consultant  Clinical geneticist  Clinical scientist  Genetic counsellor  Midwife | **Leicester (East)**  **Birmingham (Central and South)**  **London North West (North Thames)** |

Note: *Overall models based on who initiates the process and the core staffing involved throughout the service*

*Models based on survey and interview findings, plus fact checking with a genetics team member from each service*
